# Leptin and Fasting Regulate Rat Gastric Glucose-Regulated Protein 58 

**DOI:** 10.1155/2011/969818

**Published:** 2011-10-30

**Authors:** Susana B. Bravo, Jorge E. Caminos, Carmen R. González, María J. Vázquez, María F. Garcés, Libia A. Cepeda, María E. R. García-Rendueles, Antonio Iglesias-Gamarra, Consuelo Gómez-Díaz, Miguel Lopez, Justo P. Castaño, Carlos Diéguez, Rubén Nogueiras

**Affiliations:** ^1^Department of Physiology, School of Medicine, Instituto de Investigaciones Sanitarias (IDIS), University of Santiago de Compostela, 15782 Santiago de Compostela, Spain; ^2^CIBER Physiopathology of Obesity and Nitrition (CIBERobn), S. Francisco s/n, 15782 Santiago de Compostela, Spain; ^3^Department of Physiology and Internal Medicine, School of Medicine, National University of Colombia, 11001000 Bogota, Colombia; ^4^Central Service of Proteomic Unity for Research (SCAI), University of Cordoba, 14071 Cordoba, Spain; ^5^Department of Cell Biology, Physiology, and Immunology, University of Cordoba and Maimonides Institute of Biomedical Research (IMIBIC), 14014 Cordoba, Spain

## Abstract

The stomach secretes a wide range of peptides with essential metabolic functions, and thereby plays an important role in the regulation of energy homeostasis. Disulfide isomerase glucose-regulated protein 58 (GRp58) is a molecular chaperone member of the endoplasmic reticulum (ER) stress signaling pathway, which is a marker for human gastric cancer. Since GRp58 seems to be regulated by a phosphorylation/dephosphorylation pattern shift, we used the 2DE gel methodology and peptide mass fingerprinting-protein identification by means of MALDI-TOF mass spectrometry. We show that gastric mucosa GRp58 is dephosphorylated by fasting, and this effect is blunted when fasted rats are treated with leptin. Furthermore, we assessed the gene expression of GRp58 under different physiological settings known to be associated with energy homeostasis (fasting, leptin treatment and leptin deficiency). We found that intraperitoneal administration of leptin increases whereas leptin deficiency decreases GRp58 mRNA levels. However, GRp58 expression remains unchanged after fasting, indicating that leptin actions on GRp58 are no direct sensitivity to fasting. Dissection of the molecular pathways mediating the interactions between ER stress-related factors and nutrient availability, as well as their target genes, may open a new avenue for the study of obesity and other metabolic disorders.

## 1. Introduction

The stomach is a central metabolic crossroad wherein numerous signals converge and depart to control nutrient intake, appetite, and general metabolic homeostasis [1]. Thus, data gleaned over the last few years have shown that the stomach plays a key role in the regulation of food intake through the secretion of peptides such as ghrelin [2]. In addition, gastric emptying through the regulation of gastric distension also influence the rate of intestinal exposure of nutrients thereby influencing the secretion of a vast array of gastrointestinal hormones which in turn also control food intake and/or energy expenditure. Therefore, the stomach has emerged as a key organ in the regulation of body weight homeostasis, and gastric surgery is at the front line of treatment of patients with morbid obesity. However, there is a general lack of knowledge on the adaptation process that take part in the stomach in relation to energy balance. We felt the critical question pending was to identify the subcellular events that take place in this tissue and its relationship to alterations in energy balance. 

Disulfide isomerase glucose-regulated protein 58 (also known as, GRp58, ERp57, ER-60, ERp60, ERp61) is a molecular chaperone member of the ER-stress signaling pathway. Specifically, GRp58 is a 58 kDa thiol-disulfide oxidoreductase protein, physiologically involved in the folding catalysts modification of disulfide bonds in glycoprotein [3, 4], and has been previously identified in the stomach as a marker for human gastric cancer [5]. Moreover, it is also highly expressed in liver, placenta, and lung, and weakly *expressed* in other tissues [4]. *In *vitro ** and *in *vivo **studies have shown that GRp58 protein acts on glycosylated substrates through the ER resident lectins-calnexin and the calreticulin system to catalyze the isomerization or exchange of disulfide bonds [6, 7]. GRp58 substrates share common properties: they are glycoprotein, heavily disulfide bonded, and likely to form nonnative disulfide bonds [6, 7]. Most of these substrates contain abundant domains of cysteine residues, which usually promote formation of the disulfide bonds during glycoprotein folding [6–8]. In line with the interrelation among metabolism, ER-stress and chaperone function, it has been shown that GRp58 expression is induced by different stress conditions, such as glucose deprivation or hypoxia [9, 10]. Furthermore, hepatic GRp58 was phosphorylated after 12-h/24-h fasting and after leptin treatment [11], and interacts with the JAK-STAT signaling pathway implicated in the intracellular signal transduction of leptin [12].

Although GRp58 is a marker for human gastric cancer [5], few studies have addressed the regulation and potential role of ER-stress, and in particular that of GRp58, in gastric function. We aimed to investigate the involvement of GRp58 in energy homeostasis. Our results demonstrate that GRp58 is expressed in rat gastric and reveal that gastric GRp58 mRNA expression and dephosphorylation are increased after leptin treatment. Therefore, our results suggest that gastric GRp58 might play an important role in the regulation of energy homeostasis.

## 2. Methods and Procedures

### 2.1. Animals

Adult male Sprague Dawley rats (250–300 g, 8–10 weeks old) were bred in the animalario General USC; University of Santiago de Compostela, Spain, and male leptin-deficient mice (8 weeks old) were purchased from Charles River, Barcelona, Spain. Animals were housed in open cages under conditions of controlled illumination (12-hour light/dark schedule), humidity, and temperature. The animals were sacrificed by decapitation in a room separate from other experimental animals in the afternoon (16:00-17:00 h). The gastric mucosa was then collected and frozen at −80°C until analysis. All experiments were conducted in accordance with the European Union Laws on protection regarding laboratory animals after previous approval by the Ethics Committee of the University of Santiago de Compostela, Spain (Permit number: PGIDIT06PXIB208063PR).

#### 2.1.1. Experimental Setting 1: Effects of Fasting

Animal groups (*n* = 8 per group) were deprived of food for 48 h while the control group was fed *ad libitum *[13]. All animals had free access to tap water. Gastric mucosa was rapidly dissected and stored at −80°C until proteomic and mRNA analyses were performed.

#### 2.1.2. Experimental Setting 2: Effects of Leptin Treatment and Leptin Deficiency

We next investigated whether leptin affects gastric mucosa GRp58 mRNA expression and modifications in the protein phosphorylation pattern shift. Rats and mice were assigned to one of the following groups (*n* = 8 per group): (a) i.p. vehicle fed *ad libitum*; (b) i.p. leptin fed *ad libitum*; (c) i.p. vehicle after 48 h fasting and (d) i.p. leptin after 48 h fasting. On the other hand, leptin-deficient animals were distributed in three groups: (a) i.p. vehicle fed *ad libitum, *(b) i.p. vehicle after 48h-fasting and (c) i.p. leptin after 48h fasting. Animals were treated with recombinant leptin (L-4146, Sigma-Aldrich) at a dose of 0.8 *μ*g/kg of body weight every 12 h for 3 days (intraperitoneal injection) as described elsewhere [13].

For the proteomic setting, we treated rats with leptin at short term: 6 h and 24 h before the sacrifice with a single intraperitoneal injection (0,8 *μ*g/kg of body weight in a volume of 0.25 mL). We again had the same 4 groups (*n* = 4 per group). The reason we carried out this short-term experiment for proteomic approaches is that the pattern shift of phosphorylation/dephosphorylation is commonly faster than changes at transcriptional level.

### 2.2. Western Blot Analysis

Western blot analysis from 1D and 2D gels were performed as described elsewhere [14]. Briefly, gastric mucosa proteins were resolved by polyacrylamide gel electrophoresis according to the method described below and transferred to nitrocellulose membrane (Hybond C-Super, Amersham Pharmacia Biotech). Membranes were incubated with primary antibodies against Erp57 (H-220; sc-28823. Santa Cruz Biotechnology, Santa Cruz, CA, USA) and alpha-tubulin (Sigma; Poole, UK). The membranes after incubation with respective secondary antibodies were probed with HRP-conjugated secondary antibodies and the signal was developed with chemiluminescence reagents (Tropix, Bedford; MA, USA) and exposed to X-ray film. All the experiments were repeated four times and a representative result is shown.

### 2.3. Two-Dimensional Electrophoresis

2DE analyses were performed using four samples per group. Gastric mucosa was lysed at 4°C using a tissue homogenizator (Omni TissueMaster-125 Homogenizers). Lysates were prepared in lysis buffer (7 M urea, 2 M thiourea, 4% CHAPS, 5 mM magnesium acetate, 20 mMTris HCl pH 8.5, 0.2% ampholytes w/v, and bromophenol trace). Samples were centrifuged at 15000 g under 4°C during 15 min to remove the debris as described previously [15]. 

First dimension isoelectric focusing was performed with the horizontal Multiphor IPGphor III system (GE Healthcare, formerly Amersham Biosciences); using 11 cm IPG strips (pH 4–7, GE Healthcare). The sample (50 *μ*g) was dissolved by soaking in 200 *μ*L of rehydration buffer (7 M Urea, 2 M thiourea, 4% CHAPS and bromophenol trace) during 8 hours. Then, it was loaded onto an 11 cm-linear range with pH 4–7 immobilized gradient strip (4 gels per experimental group and each one originated from different animals) and isoelectric focusing was performed as recommended by the manufacturer, ramping the voltage subsequently applied to reach 6000 Vht. The strips containing focused proteins were thawed, equilibrated, and reduced in equilibration buffer (6 M urea, 50 mM M Tris-Cl pH 8.8, 2% SDS, 30% glycerol, and 2% (w/v) DTT) during 15 min, then alkylated in equilibration buffer (6 M urea, 50 mM Tris-Cl pH 8.8, 30% glycerol, 2% SDS, 2.5% (w/v) iodoacetamide. Equilibrated strips were transferred to a SDS-PAGE gel (10%), secured in place by molten agarose and subjected to electrophoresis (Mini-Protean Tetra Cell; Bio-Rad Laboratories). Afterwards, electrophoresis gels were stained using PlusOne Silver Staining Kit, Protein (GE Healthcare), as recommended by the manufacturer protocols.

### 2.4. MALDI-TOF/TOF Mass Spectrometry and Protein Identification

Spots were excised automatically in a ProPic station (Genomic Solutions, UK) and digested with modified porcine trypsin (sequencing grade; Promega), by using a ProGest digestion station (Genomic Solutions, U.K.). The gel specimens were destained twice over 30 min at 37°C with 200 mM ammonium bicarbonate/40% acetonitrile. Gel pieces were then subjected to three consecutive dehydratation/rehydratation cycles with pure acetonitrile and 25 mM ammonium bicarbonate in 50% acetonitrile, respectively, and finally dehydrated for 5 min with pure acetonitrile and dried out over 4 h at room temperature. Then, 20 *μ*L trypsin, at a concentration of 12.5 ng/*μ*L in 25 mM ammonium bicarbonate was added to the dry gel pieces and the digestion proceded at 37°C for 12 h. Peptides were extracted from gel plugs by adding 1 *μ*L of 10% (v/v) trifluoracetic acid (TFA) and incubating for 15 min. Then, they were desalted and concentrated by using *μ*C-18 ZipTip columns (Millipore) in a ProMS station (Genomic Solutions, U.K.) and directly loaded onto the MALDI plate using *α*-cyano hydroxycinnamic acid as the matrix. 

Mass analysis of peptides of each sample were performed with a MALDI-TOF/TOF (4700 Proteomics Analyzer, Applied Biosystems, USA) in automatic mode with the following setting: for the MS data, m/z range 800 to 4000 with an accelerating voltage of 20 kV and delayed extraction, peak density of maximum 50 peaks per 200 Da, minimal S/N ratio of 10 and maximum peak at 65. Spectra were internally calibrated with peptides from trypsin autolysis (M + H+ = 842.509, M + H+ = 2211.104).

For the MS/MS data, mass range was set between 60 Da and 10 Da below each precursor mass, with a minimum S/N ratio of 5, a maximum number of peak set at 65 and peak density of maximum 50 peaks per 200 Da. Proteins were assigned identification by peptide mass fingerprinting and confirmed by MS/MS analysis. Mascot 2.0 search engine (Matrix Science, U.K.) was used for protein identification running on GPS software (Applied Biosystems) over the National Center for Biotechnology Information (NCBI) protein database (updated monthly).

Search setting allowed one missed cleavage with the selected trypsin enzyme, an MS/MS fragment tolerance of 0.2 Da and a precursor mass tolerance of 100 ppm. Proteins with a statistically significant (*P* < 0.05) were positively assigned identification after considering Mr and pI values.

### 2.5. RNA Isolation and GRp58 mRNA Expression Analysis by RT-PCR

Total RNA from gastric mucosa was extracted using Trizol (Invitrogen, Inc.) according to the manufacturer's instructions. RNA was reverse transcribed and the resulting cDNA was synthesized from 2 ug total RNA by random priming RT. The resulting cDNA was subjected to PCR amplification as described at [13, 16] using sense and antisense primers specific for the rat GRp58 and the housekeeping gene hypoxanthine guanine phosphoribosyltransferase (HPRT) mRNAs (Table 1).

### 2.6. Real-Time Semiquantitative RT-PCR

For real-time semiquantitative analysis we used the Applied Biosystems 7500 Real-Time PCR System (Applied Biosystems, CA, USA), as previously described [13–15]. Rat GRp58 and 18S ribosomal rRNA relative gene expression quantification was made using specific primers and TaqMan fluorescent probes for these target genes, and the sequences are described in Table 1.

### 2.7. Statistical Analysis

The results are shown as the means ± SEM (standard error of the mean) and analyzed by using GraphPad Instat 3.05 (GraphPad Software, Inc.) software. Statistical significance was determined by Student's* t-test* when two groups were compared. In the experiments constituted by three groups the data were analyzed by one-way ANOVA followed by a post hoc multiple comparison test (Bonferroni test). *P* < 0.05 was considered significant.

## 3. Results

### 3.1. Rat Gastric GRp58 mRNA Expression

**Figure 1:**
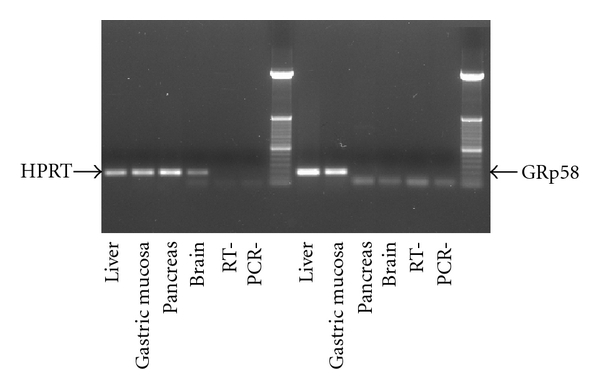
Expression of GRp58 gene in rat gastric. Representative RT-PCR assay of the expression levels of GRp58 mRNA in liver and gastric mucosa whereas pancreas and brain failed to show any amplification. HPRT was used as a housekeeping gene.

Expression of the mRNA encoding GRp58 gene (Figure 1) was evaluated by RT-PCR and compared to housekeeping gene HPRT. This analysis demonstrates the expression of the message of the gene in male rat liver and gastric mucosa whereas its expression was not found in tissues such as pancreas and brain (Figure 1). 

### 3.2. Rat Gastric GRp58 Protein Levels

**Figure 2:**
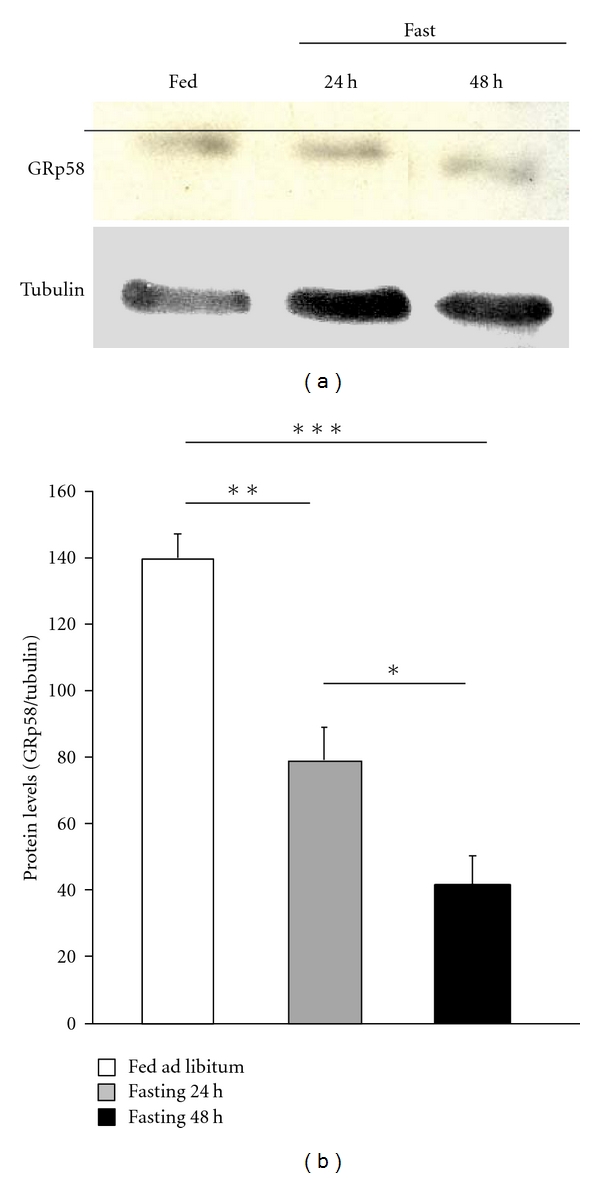
Representative Western blot analysis (a) and quantification (b) of rat gastric GRp58 protein levels in fasted rats. Fifty micrograms of total proteins were loaded on a 10% SDS-PAGE gel. To confirm equal loading, the same blot was stripped off and incubated with monoclonal beta-tubulin antibody. Values are mean ± SEM of 8 rats per group.

After discovering that GRp58 is expressed in the gastric mucosa by means of RT-PCR, we employed Western blot analysis to determine whether the message is translated into protein. Using a specific primary antibody for GRp58, we detected a major band in protein extracts of rat gastric, and observed that protein levels of GRp58 were decreased after 24 h and 48 h of fasting (Figure 2). Moreover, in the gastric mucosa of rats fasted during 48 h, we found that the band had a lower molecular weight, likely suggesting a dephosphorylation (Figure 2). 

### 3.3. Proteomic Identification and Molecular Analysis

**Figure 3:**
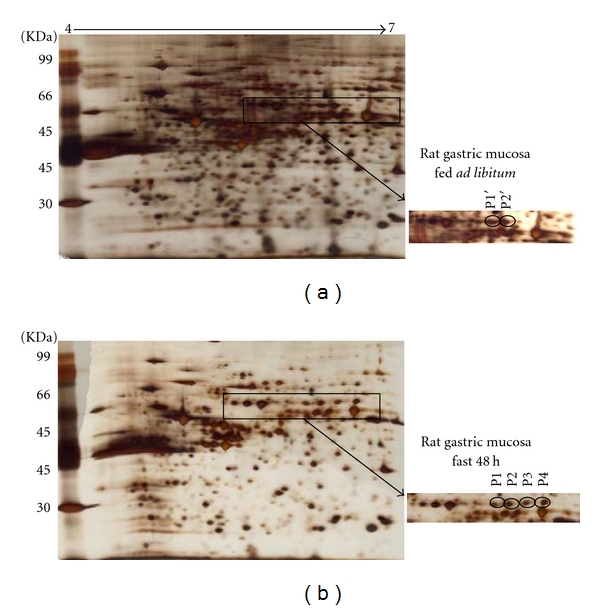
Representative 2-DE gel image of gastric mucosa proteins from fed *ad libitum* (a) and 48 h fasted (b) rats. Gastric proteins were extracted from mucosa and separated on an immobilized pH 4–7 nonlinear gradient strip followed by separation on a 12% polyacrylamide gel. Silver stained gel and spots differentially expressed proteins in fed controls relative to fasted rats were picked, and analysis by mass spectrometry allowed the detection and identification of different GRp58 phosphorylated forms. Values are mean ± SEM of 8 rats per group.

**Figure 4:**
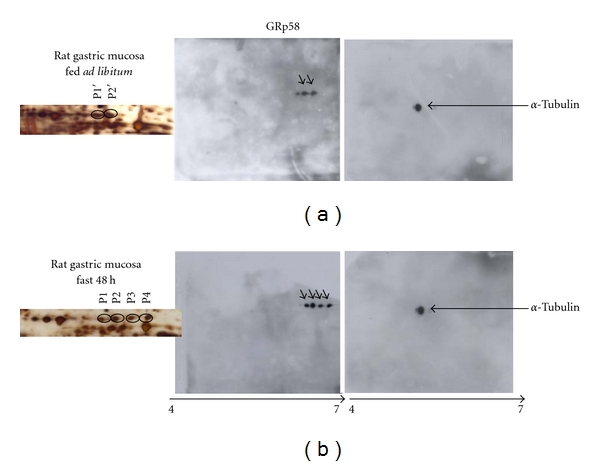
2DE gel electrophoretic analysis and immunoblot image of gastric mucosa proteins from fed (a) and 48 h fasted (b) rats. Image shows the position of GRp58 phosphorylated forms and alpha-tubulin detected by incubation with anti-GRp58, and anti alpha-tublin, respectively. The position of GRp58 phosphorylated forms and alpha-tubulin in the silver-stained and nitrocellulose 2D is indicated by arrows.

To test the hypothesis that GRp58 was dephosphorylated during fasting, we performed proteomic analysis. Representative 2-DE gel images of rat gastric tissue with differential expressed protein in fed state (Figure 3(a)) compared to fasted state (Figure 3(b)). Differentially expressed protein spots (marked with letters) were picked, digested with trypsin, and analyzed by peptide mass fingerprinting as previously described (Tables 2 and 3) [17, 18]. The peptide mass fingerprints were acquired by mass spectrometry using a matrix-assisted laser desorption/ionization time-of-flight (MALDI-TOF). All analyzed spots correspond to multiple phosphorylated forms of GRp58 protein, which were identified by the comparison of peptide mass fingerprint data generated by MALDI-TOF mass spectrometry against theoretically digested *Homo sapiens*, Swiss-Prot and TrEMBL database, and by the use of Mascot software. Among the identified protein spots, a 4-protein series with identical molecular weight of approximately 58 kDa and different isoelectric points were identified as GRp58. These differences reflect different degrees of phosphorylation as previously described [19–21]. The identified *phosphorylated forms* of GRp58 protein in rat gastric along with referred data are listed in Table 3. *These phosphorylation profile shifts were identified through a *2-DE Western blot analysis using an anti-GRp58 antibody, recognizing four immunoreactive protein spots *(Figures 4(a) and 4(b)) that showed a basic shifted signal in the profile of GRp58.*

### 3.4. Fasting Induces GRp58 Dephosphorylation Whereas Leptin Blunts Fasting-Induced Dephosphorylation of Gastric Mucosa GRp58

**Figure 5:**
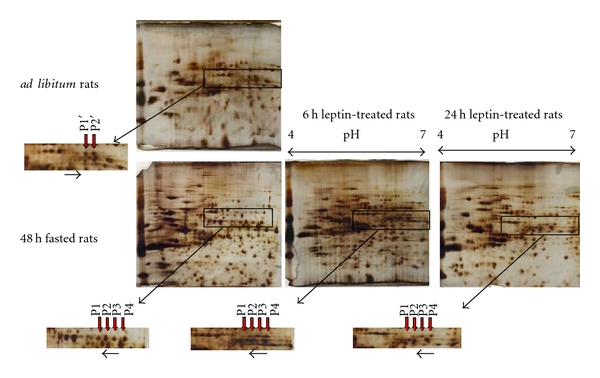
2-DE gel image of GRp58 phosphorylation pattern of rat gastric from fed, 48 h fasted rats, and 48 h fasted rats treated with leptin and sacrificed after 6 h and 24 h of leptin administration. GRp58 protein acidic isoelectric points decreased in 2DE gels in fasted and leptin treated rats compared to vehicle-treated control fed rats. Gastric mucosa proteins were extracted and separated on an immobilized pH 4–7 non-linear gradient strip followed by separation on a 12% polyacrylamide gel. Red arrows show the different phosphorilation pattern observed in different treatments.

Additionally, using 2DE, we found that 48 h of fasting dephosphorylated gastric mucosa GRp58 in comparison with rats fed *ad libitum* (Figure 5). When 48 h fasted-rats were treated with leptin for 6 hours or 24 h, we observed that the fasting-induced GRp58 dephosphorylation was blunted (Figure 5). Therefore, our results indicate that the dephosphorylation of gastric mucosa GRp58 induced by fasting, a hypoleptinemic state, is likely mediated by circulating leptin levels. 

### 3.5. Gastric Mucosa GRp58 mRNA Levels Are Induced by Leptin

**Figure 6:**
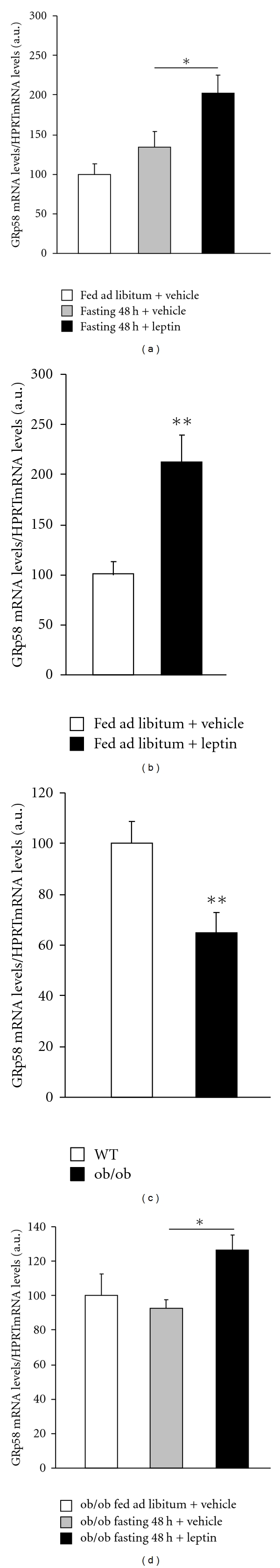
Gastric mucosa GRp58 mRNA levels in fed, 48 h fasted rats, and 48 h fasted rats treated with leptin (a). Gastric mucosa GRp58 mRNA levels in fed versus leptin-treated rats (b). Gastric mucosa GRp58 mRNA levels in wild-type mice versus leptin-deficient mice (ob/ob) (c). Gastric mucosa GRp58 mRNA levels in ob/ob mice fed *ad libitum*, fasted for 48 h, and fasted for 48 h treated with leptin (d). *n* = 8 per group. Gastric mucosa GRp58/18S were measured using RT-real time PCR. Mean ± SEM are reported. *P < 0.05, **P < 0.01.

We then measured the mRNA expression of gastric mucosa GRp58 in the same experimental paradigm described above, meaning, fed ad libitum, fasted, and fasted leptin-treated rats. We failed to detect any significant change in the gene expression of GRp58 of fasted rats when compared to feed ad libitum rats (Figure 6(a)). However, we found that leptin treatment was able to trigger gastric mucosa GRp58 mRNA levels in fasted rats in comparison with vehicle-treated rats (Figure 6(a)). Similar results were detected in fed *ad libitum* rats, which showed a clear upregulation in GRp58 gene expression after the leptin treatment (Figure 6(b)). According to pharmacological data, the endogenous lack of leptin downregulated gastric mucosa GRp58 gene expression when compared to wild-type mice (Figure 6(c)). Finally, when leptin-deficient mice were fasted for 48 h we failed to detect changes in GRp58 mRNA levels, but when those fasted mice were treated with exogenous leptin, an increase in gastric mucosa GRp58 gene expression was found (Figure 6(d)). Overall, our findings show that leptin is an important mediator of GRp58 gene expression in the gastric mucosa.

## 4. Discussion

The endoplasmic reticulum (ER) is a multifunctional organelle that plays a critical role in multiple cellular processes including, among others, protein and lipid biosynthesis, steroid production, calcium homeostasis, and carbohydrate metabolism [6]. In addition, the ER contains a large number of calcium-dependent molecular chaperones and folding enzymes, which are physiologically responsible for several cotranslational and posttranslational modifications [22, 23]. Alterations in energy metabolism including glucose/energy deprivation, oxidative injury, hypoglycemia, hypoxia, and high-fat diet impair ER homeostasis. More specifically, nutritional excess induces ER stress in subcutaneous adipose tissue of obese human subjects [24]. Additionally, ER stress has been related to the development of atherosclerosis and diabetes [24–27]. For instance, obese humans and rodents develop ER stress in liver and adipose tissues, leading to insulin resistance whereas weight-loss decreases ER stress and improves insulin sensitivity, suggesting a correlation between ER stress and the metabolic syndrome [27, 28]. 

By using proteomics analysis, we found evidence of a GRp58 phosphorylation/dephosphorylation pattern shift in the rat gastric under changes in nutritional status. More specifically, using the 2DE gel methodology and peptide mass fingerprinting-protein identification by means of MALDI-TOF mass spectrometry, we show that gastric mucosa GRp58 is dephosphorylated by fasting, and this effect is blunted when fasted rats are treated with leptin. GRp58, a well-known stress protein, has been previously identified in the stomach as a marker for human gastric cancer [5]. Furthermore, previous works have demonstrated the presence of leptin and leptin receptor in the stomach, suggesting that gastric mucosa cells may be targets for leptin [29, 30]. GRp58 and signal transducer and activator of transcription 3 (STAT3), an essential signaling molecule mediating the biological actions of leptin [31], have been localized in both the cytoplasm and nucleus [32, 33]. In this regard, some studies have shown that GRp58 may be implicated in the transcriptional control of STAT3 [12, 32]. Notwithstanding the evidence mentioned above, there was a general lack of knowledge regarding a potential role of gastric GRp58 in metabolism. Concurring with a role of gastric GRp58 on energy balance, we demonstrate that leptin induces, whereas leptin-deficiency reduces, gastric mucosa GRp58 mRNA expression, suggesting that leptin modulates both GRp58 phosphorylation and expression. Since these effects seem to be nutritional, it is plausible to hypothesize that other tissues that are target of leptin action in nutritional context like the intestine might be also involved in the regulation of GRp58. For instance, leptin is expressed and secreted by inflamed colonic epithelial cells [34], it may act as a growth factor in colon tissue [35]. Moreover, leptin receptors have been located in the rat colonic epithelium [36]. Thus, the role of leptin on GRp58 throughout the entire gastrointestinal tract deserves further investigation. 

Our findings obtained in rat gastric differ from data obtained in the rat liver, where it was found that serine 150 of GRp58 was phosphorylated by both fasting and leptin [11]. Thereby, those results were indicating that nutritional-induced changes in liver GRp58 phosphorylation were not mediated by circulating leptin levels. Our current data demonstrating that leptin blocks the fasting-induced dephosphorylation of gastric GRp58 suggest that changes in the activity of this protein are modulated by nutritional status. Importantly, leptin seems to be responsible for those nutritional-induced changes in gastric GRp58 phosphorylation. Moreover, the different results obtained in the pattern of GRp58 phosphorylation in the liver [11] and the stomach suggests that GRp58 is regulated by nutritional status in a tissue-specific manner. Since we have detected multiple phosphorylated forms of GRp58 protein in the gastric mucosa (Figure 2), we hypothesize that these phosphorylated forms could contribute to the tissue-specific regulation of GRp58 in response to fasting and leptin treatment. 

It is important to point out several issues that deserve further attention. First, the functional role of phosphorylation/dephosphorylation of GRp58 caused by physiological and physiopathological conditions is largely unknown. Second, the sites of phosphorylation/dephosphorylation of Grp58 are different in each tissue [11, 37], so it might be possible that the GRp58 phosphorylated forms that we detected in the gastric mucosa also have different phosphorylation sites. Third, the factors that induce these phosphorylation/dephosphorylation shifts in a tissue-specific manner are mostly unknown. All these issues seem relevant to elucidate the precise role and mechanisms mediating GRp58 actions in the organism. 

In addition to the actions of nutritional status on the pattern of gastric mucosa GRp58 phosphorylation, we have also assessed the regulation of GRp58 gene expression. Our findings indicate that leptin increases GRp58 mRNA expression whereas leptin deficiency decreases the levels of this protein. Since leptin lowers blood glucose levels and leptin-deficient mice are hyperglycemic, our results are in agreement with previous in vitro results indicating that GRp58 expression is induced by glucose deprivation [38]. 

Although GRp58 mRNA expression remained unchanged after fasting, we found that its protein levels were decreased after 24 h and 48 h of fasting. These results suggest that fasting modulates GRp58 at post-transcriptional levels. Therefore, it is likely that the actions of leptin on GRp58 are dependent of nutritional status. On the other hand, the ingestion of specific components of the diet like fructose increases the secretion of gastric leptin [39], and this might also affect GRp58 gastric levels.

Taken together, our findings obtained on the dephosphorylation and total mRNA expression of GRp58 indicate that leptin regulates the phosphorylation and expression of gastric GRp58 in rats. It also seems reasonable to hypothesize that at least some of the actions of leptin at the gastric level might be mediated by GRp58 [40].

In conclusion, our results demonstrate that GRp58 phosphorylation responds rapidly to changes in dietary energy and leptin treatment, and thereby support that GRp58 can play an important physiological role in the signaling pathways related to energy balance in the stomach. More precisely, our findings indicate that (a) fasting dephosphorylates gastric mucosa GRp58, (b) leptin blunts fasting-induced GRp58 dephosphorylation, and (c) leptin stimulates gastric mucosa mRNA expression. Dissection of the molecular pathways mediating the interactions between ER stress-related factors and nutrient availability, as well as their target genes may open a new avenue for the study of obesity and other metabolic disorders. 

## Figures and Tables

**Table 1:** PCR primers and probe sequence for rat GRp58 and 18S ribosomal rRNA.

Primer/probe	Sequence	Genebank accession number
GRP58FW	5′-GGACCAGCTTCAGTTCCTCTCA-3′	NM_017319.1
GRP58RV	5′-TGCTGGCTGCTTTTAGGAACTC-3′	
GRP58Pb	FAM 5′-ATGCCTCGGTGGTGGGCTTTTTCA-3′TAMRA	

18SFW	5′-CGGCTACCACATCCAAGGAA-3′	M11188.1
18SRV	5′-GCTGGAATTACCGCGGCT-3′	
18SPb	FAM 5′-GACGGCAAGTCTGGTGCCAGCA-3′TAMRA	
HPRT FW	5′-CAGTCCCAGCGTCGTGATTA-3′	NM_012583
HPRT RV	5′-AGCAAGTCTTTCAGTCCTGTC-3′	

**Table 2:** Cutoff score value for protein: 71 (score is −10∗Log(*P*), where *P* is the probability that the observed match is a random event. Protein scores greater than 71 are significant (*P* < 0.05)). The software used for peak-picking was 4000 Series Explorer (TM) RAC Software, version 3.5.3 (Applied Biosystems/MDS SCIEX, Concord, Ontario, Canada). Parameters and thresholds used for peak-picking: (a) intensity or S/N threshold: S/N = 10, local noise window width (m/z) = 250, min peak width at full width half max (bins) = 2.9; (b) means of calibrating each spectrum: internal calibration with peptides from trypsin autolysis (M + H^+^ = 842.509, M + H^+^ = 2211.104); (c) resolution: 12000 for the mass 842.51 and 18000 for the mass 2211. Search parameters: (a) MASCOT (Matrix Science, London, UK) software, VERSION 2.0; (b) enzyme specificity: trypsin; (c) missed cleavages permitted: 1; (d) fixed modification (s): carbamidomethyl (C); (e) variable modifications: oxidation (M), Phospho (ST), Phospho (Y); (f) mass tolerance for precursor ions: ±100 ppm; (g) mass tolerance for fragment ions: ±0.2 Da; (h) name of database searched and release version: NCBInr 20080628 (6655203 sequences); (i) species restriction: Mammalia (mammals) (689751 sequences); (j) acceptance criteria: cut-off score value for protein: 71 (score is -10∗Log(*P*), where *P* is the probability that the observed match is a random event. Protein scores greater than 71 are significant (*P* < 0.05)).

Spot No	protein score	protein score 1%	Coverage %	Number of peptides identified
P1′	170	100	60	13
P2′	149	100	50	12
P1	282	100	90	24
P2	439	100	95	24
P3	288	100	64	15
P4	237	100	55	12

**Table 3:** Identification of GRp58 protein in the gastric mucosa of the rat. Rat gastric proteins were separated by 2-DE and identified by means of MALDI-TOF. The protein identified represents the spots shown in Figures 1(a) and 1(b): protein disulfide-isomerase A3 precursor (Disulfide isomerase ER-60; ERp60; 58 kDa microsomal protein; p58; ERp57; HIP-70; Q-2; 35 petides), *the spots labelled as P1′ and P2′ are the same spots as P1 and P2 but in different gels. *

Protein MW: 57043.9 Protein PI: 5.88 Accession No: gi|1352384|						
Observed Mr (expt)	Mr (calc)	± da	± ppm	Start-end	Sequence	Spot number

823.4785	823.4559	0.0226	27	190–196	IVAYTEK	p1′,p1
866.4574	866.4617	−0.0043	5	387–393	YKELGEK	p1′,p1
877.4915	877.489275	0.0025	3	268–275	LNFAVASR	p1′,p1
995.5628	995.5632	−0.0004	0	102–111	QAGPASVPLR	p1′,p1
997.5101	997.459	−0.0511	−51	153–161	DASVVGFFR	p2
1084.5674	1084.5688	0.0014	1	95–104	YGVSGYPTLK	p2′,p1,p2,P3
1123.6582	1123.6589	0.0007	1	101–111	KQAGPASVPLR	p1′,p1
1125.547	1125.5436	0.0034	3	243–251	NTKGSNYWR	p1
1172.5404	1172.4702	−0.0702	−60	336–344	FVMQEEFSR	p1,p2
1179.5865	1179.6239	0.0374	32	174–183	AASNLRDNYR	p2′,p1,p2
1188.5354	1188.5305	−0.0049	−4	344–336	FVMQEEFSR	p2′,p1,p2,P3
1191.6005	1191.6084	0.0079	7	63–73	LAPEYEAAATR	p1′,p2′,p1,p2,P3,P4
1236.5127	1236.5029	−0.0098	−8	108–119	DGEEAGAYDGPR	p2′,p1,p2,P3
1244.6633	1244.6276	−0.0357	−29	184–194	FAHTNVESLVK	p1,P3
1341.6837	1341.683	−0.0007	−1	449–460	GFPTIYFSPANK	p2′,p1,p2,P3,P4
1347.7043	1347.7015	0.0028	2	33–44	RLAPEYEAAATR	p1
1373.6736	1373.5839	−0.0897	−65	352–362	FLQEYFDGNLK	p2
1394.6587	1394.5729	−0.0858	−62	162–173	DLFSDGHSEFLK	p1,p2
1396.6954	1396.6727	−0.0227	−16	367–379	SEPIPETNEGPVK	p2′,p1,p2,P3,P4
1397.5784	1397.7063	0.1279	92	83–94	VDCTANTNTCNK	p1′,p2′,p2,P3,P4
1397.7059	1397.7063	0.0004	0	472–482	ELNDFISYLQR	p1′,p2′,p1,P3,P4
1469.7787	1469.691	−0.0877	−60	449–461	GFPTIYFSPANKK	p1,p2,P4
1472.6838	1472.6184	−0.0654	−44	336–347	FVMQEEFSRDGK	p2
1488.6787	1488.6698	−0.0089	−6	336–347	FVMQEEFSRDGK	P3
1529.7747	1529.775	0.0003	0	352–363	FLQEYFDGNLKR	p1′,p2′,p1,p2,P3,P4
1593.8483	1593.8551	0.0068	4	483–496	EATNPPIIQEEKPK	p2′,p1,p2,P3,P4
1607.7476	1607.764	0.0164	10	259–271	DLLTAYYDVDYEK	p1′,p2′,p2,P3,P4
1636.7523	1636.6552	−0.0971	−59	434–448	MDATANDVPSPYEVK	p1,p2,P4
1652.7472	1652.6615	−0.0857	−52	434–448	MDATANDVPSPYEVK	p2
1652.7662	1652.7704	0.0042	3	105–119	IFRDGEEAGAYDGPR	p1′,p2′,p1,p2,P3,P4
1744.8864	1744.7698	−0.1166	−67	131–146	QAGPASVPLRTEDEFK	p2
1746.9286	1746.8311	−0.0975	−56	289–304	TFLDAGHKLNFAVASR	p1′
1800.9377	1800.9132	−0.0245	−14	364–379	YLKSEPIPETNEGPVK	p1′,p2′,p1,p2,P3,P4
1950.9331	1950.7816	−0.1515	−78	259–274	DLLTAYYDVDYEKNTK	p2
2463.1279	2462.9673	−0.1606	−65	83–104	VDCTANTNTCNKYGVSGYPTLK	p2
